# Geometric deep learning for diffusion MRI signal reconstruction with continuous samplings (DISCUS)

**DOI:** 10.1162/imag_a_00121

**Published:** 2024-04-02

**Authors:** Christian Ewert, David Kügler, Rüdiger Stirnberg, Alexandra Koch, Anastasia Yendiki, Martin Reuter

**Affiliations:** German Center for Neurodegenerative Diseases (DZNE), Bonn, Germany; A. A. Martinos Center for Biomedical Imaging, Massachusetts General Hospital, Boston, MA, United States; Department of Radiology, Harvard Medical School, Boston, MA, United States

**Keywords:** deep learning, geometric deep learning, denoising, diffusion MRI, signal reconstruction, *q*-space

## Abstract

Diffusion-weighted magnetic resonance imaging (dMRI) permits a detailed in-vivo analysis of neuroanatomical microstructure, invaluable for clinical and population studies. However, many measurements with different diffusion-encoding directions and possibly *b*-values are necessary to infer the underlying tissue microstructure within different imaging voxels accurately. Two challenges particularly limit the utility of dMRI: *long acquisition times* limit feasible scans to only a few directional measurements, and the *heterogeneity of acquisition schemes across studies* makes it difficult to combine datasets. Left unaddressed by previous learning-based methods that only accept dMRI data adhering to the specific acquisition scheme used for training, there is a need for methods that accept and predict signals for arbitrary diffusion encodings. Addressing these challenges, we describe the first geometric deep learning method for *continuous* dMRI signal reconstruction for arbitrary diffusion sampling schemes for both the input and output. Our method combines the reconstruction accuracy and robustness of previous learning-based methods with the flexibility of model-based methods, for example, spherical harmonics or SHORE. We demonstrate that our method outperforms model-based methods and performs on par with *discrete* learning-based methods on single-, multi-shell, and grid-based diffusion MRI datasets. Relevant for dMRI-derived analyses, we show that our reconstruction translates to higher-quality estimates of frequently used microstructure models compared to other reconstruction methods, enabling high-quality analyses even from very short dMRI acquisitions.

## 1 Introduction

Magnetic Resonance Imaging (MRI) has long been a key technology for the in-vivo study of the human brain in health and disease. Diffusion-weighted MRI (dMRI), in particular, provides “invaluable information” (Tuch, 2004) on tissue microstructure and macro-anatomy. For example, dMRI acquisitions enabled the study of neuroinflammatory response and structural breakdown in Alzheimer’s disease (Fick et al., 2017) as well as structural remodeling after stroke (Galazzo et al., 2018). Beyond the study of local microstructure, high-quality diffusion acquisitions yield reconstructions of the brain’s neural pathways facilitating the study of brain architecture and connectivity.

However, constraints related to acquisition time and signal-to-noise ratio impose trade-offs in image-space and q-space (the space representing the direction and magnitude of diffusion weighting). Furthermore, different approaches to analyzing dMRI data (Wedeen et al., 2005; H. Zhang et al., 2012) recommend different acquisition schemes. As a result, different studies have different acquisition protocols (single-, multi-shell, or grid-based sampling), where q-space is sampled at different sets of directions and b-values. In this paper, we focus on q-space super-resolution, that is, from a limited series of *obtained measurements* (**observations**), we predict the *unknown signals* for additional diffusion vectors in continuous q-space (**query vectors**). We present a method allowing for flexible input from and flexible query for any q-space acquisition scheme. This method reliably infers additional signals and is a prerequisite for data harmonization, for example, by resampling datasets acquired with different q-space samplings onto an arbitrary common sampling scheme, addressing both of the challenges mentioned above.

Learning-based methods have shown impressive success in q-space super-resolution and, despite only utilizing relatively simple neural networks (Gibbons et al., 2019; Golkov et al., 2016; Li et al., 2021; Lin et al., 2019; Nath et al., 2019, 2020; Ye, 2017), have outperformed prior model-based methods (Descoteaux et al., 2007; Özarslan et al., 2009, 2013). Generally, we distinguish between discrete and continuous reconstruction models. *Output-continuous* methods permit the flexible signal reconstruction for arbitrary q-vectors whereas *output-discrete* methods are restricted to an a priori fixed choice of q-vectors. Similarly, *input-continuous* methods can accept any q-space acquisition scheme, whereas *input-discrete* methods are inherently tied to a fixed acquisition scheme and do not apply to other acquisition schemes. The denoising and super-resolution capabilities of learning-based models are proven across medical imaging settings (Gondara, 2016). However, so far, proposed learning-based methods for dMRI signal reconstruction are from one of four categories: 1. both input- and output-discrete (Gibbons et al., 2019; Golkov et al., 2016; Li et al., 2021), 2. input-discrete (with Graph-CNN) (Chen et al., 2020, 2022), 3. achieve continuity through fitting a continuous model representation (Lin et al., 2019; Nath et al., 2019, 2020), and 4. are only output-continuous (Ren et al., 2021). In contrast to learning-based methods, model-based methods rely on assumptions about the physics of the dMRI signal and were proposed for both discrete (Paquette et al., 2015) and continuous signal reconstruction (Descoteaux et al., 2007; Fick et al., 2016; Özarslan et al., 2009, 2013), but can lack robustness when only few, noise-afflicted measurements (from a short acquisition) are available.

*Di*ffusion MRI *S*ignal Reconstruction with *C*ontin*u*ous *S*amplings (DISCUS) combines the advantages of the continuous reconstruction of model-based methods with the improved prediction and denoising capabilities of deep learning models. Instead of relying on a generative model of the signal, or decomposing the signal in terms of continuous basis functions, we interpret the reconstruction task as the continuous completion of a finite point cloud and learn to perform this task in an entirely data-driven fashion building on geometric deep learning methods for point clouds. Our contributions are:

- We provide the first learning-based *input-* and *output-continuous* method for continuous reconstruction of q-space signals that can be initialized with any (sufficiently dense) q-space sampling scheme.
- We demonstrate that our method outperforms model-based methods and performs on par with discrete learning-based methods on single-, multi-shell, and grid-based dMRI datasets while adding sampling flexibility.
- We show that our reconstructed signals yield better estimates of signal-derived microstructure models than those reconstructed with other methods, for example, for *Diffusion Tensor Imaging* (DTI), *Diffusion Kurtosis Imaging* (DKI), *Neurite Orientation Dispersion and Density Imaging* (NODDI), and *Mean Apparent Propagator MRI* (MAP-MRI).

## 2 Problem Statement and Related Work

Diffusion-weighted MRI reconstruction aims to infer additional signals from a set of acquired measurements. For clarity, we use the following definitions and concepts: A *dMRI acquisition* consists of a paired series of *diffusion vectors* and *diffusion-weighted images (DWI)*. Each DWI is a 3D MR image and the diffusion vector is an a priori chosen parameter vector (updated in acquisition) determining the direction and weighting of the diffusion measurement. This vector is also referred to as the q-vector drawn from $q\text{-space}\subset {\mathbb{R}}^{3}$. At a fixed spatial position, the dMRI acquisition is a paired series $\left(q_{i},s_{i}\right)$ of q-vectors $q_{i}$ and scalar diffusion signals $s_{i}$. In the reconstruction context, we refer to this paired series as the *observation set* $x_{obs}$ . Based on the observation set, we aim to predict the missing signal $\hat{s}$ corresponding to the *query vector* q, that is, obtain $\hat{s}=ℳ(q|x_{obs})$ with the reconstruction model ℳ. Generally, q and $\hat{s}$ may also be lists of arbitrary size.

The q-space super-resolution problem, that is, the prediction of signals for additional q-vectors, stands in contrast to image super-resolution (Albay et al., 2018; Chatterjee et al., 2021), where additional signals for new spatial locations are predicted. As previously introduced, we differentiate between discrete (Chen et al., 2020, 2022; Gibbons et al., 2019; Golkov et al., 2016; Li et al., 2021; Paquette et al., 2015) and continuous (Cheng et al., 2013; Descoteaux et al., 2007; Fick et al., 2016; Jha et al., 2022; Lin et al., 2019; Mani et al., 2022; Michailovich et al., 2011; Nath et al., 2020, 2019; Özarslan et al., 2009, 2013; Rathi et al., 2014; Sedlar et al., 2021; Ye, 2017) q-space super-resolution methods. *Input-discrete* methods can only be applied to the specific acquisition scheme of the training data. Similarly, *output-discrete* methods can only predict signals for specific q-vectors used during training. Because of this, the data from multiple studies cannot be mixed in training to improve generalization and offset the cost of training data acquisition for input- or output-discrete methods. In fact, input-discrete models trained on data with a particular sampling scheme cannot be applied to datasets acquired with different sampling schemes. On the other hand, *input-continuous* models are applicable to any acquisition scheme and *output-continuous* models permit the flexible signal prediction for arbitrary q-vectors given the information of the observation set.

Reconstruction methods follow either a *learning-* or a *model-based* paradigm. Learning-based methods require training on a collection of data *across* voxels while model-based methods rely on a diffusion signal model fit to the observation set of the particular voxel. Model-based methods can lack robustness when measurements of only a few q-space points are available. In contrast, learning-based methods have proved their denoising capabilities in various settings (Fadnavis et al., 2020; Gondara, 2016; Koonjoo et al., 2021). An example of an entirely discrete, model-based method is compressed sensing for diffusion spectrum imaging (DSI) (Paquette et al., 2015), which uses the Fourier relationship between q-space and the propagator space for reconstruction. Entirely continuous (input- and output-continuous) model-based methods decompose the signal in terms of continuous basis functions such as spherical harmonics or spherical ridgelets for spherical signals (Descoteaux et al., 2007; Michailovich et al., 2011) and SHORE, MAP-MRI, spherical ridgelets with radial decay, and other approaches for 3D signals (Cheng et al., 2013; Özarslan et al., 2009, 2013; Rathi et al., 2014).

Entirely discrete, learning-based methods predict diffusion signals or derived quantities based on the observation set for individual voxels, for example, FA and MD from DTI or NDI and ODI from NODDI. They employ linear (Varadarajan & Haldar, 2018) or non-linear models, that is, multi-layer perceptrons (Golkov et al., 2016; Grussu et al., 2020), or predict whole images using convolutional neural networks (CNN) (Gibbons et al., 2019; Li et al., 2021).

So far, almost all proposed learning-based methods achieve entirely continuous reconstruction only indirectly by the workaround of incorporating established models, thus inheriting their limitations. Instead of learning reconstruction end-to-end, these methods first decompose the signal in terms of basis functions like spherical harmonics (Lin et al., 2019; Jha et al., 2022; Nath et al., 2020; Mani et al., 2022) or SHORE functions (Nath et al., 2019; Ye, 2017), and train inherently discrete networks to predict coefficients of basis functions in the target domain (for signal reconstruction) or diffusion signal-based microstructure indices from coefficient vectors in the source domain. These methods are limited by a loss of information during the conversion to basis coefficients in the source and target domains. Furthermore, an optimal order truncating the basis of these models may depend on the tissue type, and assumptions about the signal properties may yield sub-optimal results if only a few noisy measurements are available, for example, the exponential signal decay in the SHORE model. In addition, learned coefficients may only be optimal for the number of observation and query vectors used during training as no work to date has trained a method with observation and query sets containing a wide range of different numbers of vectors. Graph-convolutional approaches interpret the q-space sampling as a graph (Chen et al., 2020, 2022), but have thus far only been applied to a priori chosen acquisition schemes and not addressed the challenges imposed by arbitrary acquisition schemes. Ren et al. (2021) proposed an output-continuous method to reconstruct DWIs in *image space* using a CNN from other MRI contrasts, that is, a stack of non-diffusion-weighted, T1-, and T2-weighted images, whereas we reconstruct DWIs in *voxel space* from other DWIs.

To our knowledge, no learning-based, input- and output-continuous method offering full flexibility for reconstruction has been proposed to date. While some methods incorporate larger spatial contexts to make predictions for a particular voxel, that is, incorporating adjacent voxel or even image slice information, model-based methods such as SHORE and spherical harmonics take q-space data only from the voxel at hand into account. To provide a fair comparison with these methods, we use the same strategy, that is, we do not incorporate neighborhood information of any kind.

## 3 Method

Our *DISCUS* method has the flexibility of learning from and predicting signals for any q-space sampling scheme. We first introduce a list of aims for our reconstruction model, then explain the architecture and augmentation scheme, followed by the loss function used for training.

First, we pose the following six aims for our method:

1a) *Flexibility to input any number of observations* and 1b) *Invariance to the choice of observation vectors:* dMRI acquisitions often define unique q-space sampling schemes containing from single-digit to several hundred measurements. 2) *Invariance to the ordering of observation vectors:* while dMRI measurements are obtained sequentially, the ordering is arbitrary and has no impact on reconstruction. 3) *Invariance to the sign of observation and query vectors:* dMRI signals are antipodally symmetric, that is, s(q)=s(−q) . 4) *Invariance to rotations of the* q*-space basis:* Since the q-space signal is reconstructed independently at each voxel (see Section 2), reconstruction relies only on the relative position of observation vectors to each other and the query vectors, not a specific choice of reference frame. 5) *Invariance to T2-shinethrough:* Each diffusion measurement has a T2-weighted and a diffusion-weighted signal component. As both components are independent per spatial voxel, the T2-weighted component has no impact on the diffusion signal reconstruction.

We address these aims with specific design choices in the architecture (requirements 1a, 2, and 3), with data augmentation (requirements 1b and 4), and preprocessing of the data (requirement 5). To isolate the diffusion signal from the T2-weighted component, that is, the signal at the origin of q-space, we simply normalize the diffusion-weighted signal by the mean q≡0 image, since the signal solely consists of the T2-weighted component there.

### 3.1 Architecture

**Fig. 1.**
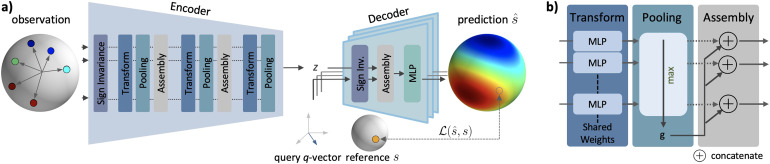
*DISCUS* architecture: Our method is designed to learn a continuous signal from a finite list of diffusion measurements end-to-end. (a) The *DISCUS* architecture consists of an encoder and a decoder. The encoder input is the paired series of q-vectors and observed signals s (the observation set $x_{obs}=[q,s]$ ). It summarizes the information into a latent representation z that is independent of the order and the specific measurements. The decoder predicts signals for arbitrary query q-vectors given the latent vector z from the encoder. The encoder and decoder start with the extraction of features invariant to the sign of the input vectors. The discrepancy between predicted signals $\hat{s}$ for query q-vectors and (withheld) corresponding reference signals s is employed as loss $ℒ(\hat{s},s)$ for training. (b) The interplay of the transform and pooling modules implements permutation invariance. The transform module transforms features individually while the pooling module aggregates information across features. Between pooling and transform modules, the assembly module concatenates features for further processing.

Our architecture features an encoder and a decoder (see Fig. 1a). Per voxel, the encoder $E:{\mathbb{R}}^{4\;\times \;N}\to {\mathbb{R}}^{L}$ receives the observation set $x_{obs}$ , that is, N diffusion measurements (q-vectors and acquired signals), as input and outputs a latent vector $z=E(x_{obs})$ summarizing the information across measurements. The purpose of the encoder is to obtain a representation characterizing the diffusion profile that is independent of the order, number, and specific choice of measurements. The decoder $D:{\mathbb{R}}^{3\;\times \;M}\times {\mathbb{R}}^{L}\to {\mathbb{R}}^{M}$ predicts the signals $\hat{s}=D(q|z)$ for the query vectors q given the latent vector z. The combination of encoder and decoder yields our reconstruction model $\hat{s}=D(q|z)=D(q|E(x_{obs}))=ℳ(q|x_{obs})$ . Here, we provide an overview of the components of our method as well as their properties. A detailed schematic of the *DISCUS* architecture can be found in Appendix Figure A.

#### 3.1.1 Encoder

The encoder’s task is to extract the relevant information for reconstruction from the N input measurements while keeping the number N flexible (aim 1a) and to implement aims 2 and 3. The N observation vectors are processed along N processing paths—one path is dynamically allocated per input observation vector. Each module works along these paths or across these paths. Along-the-paths modules apply a single function independently to each path. In contrast, the across-the-paths modules merge information between these paths (Qi et al., 2017). The observation vectors are processed by the sign-invariance module followed by a sequence of alternating transform and pooling modules with interleaved assembly modules (see Fig. 1a, “Encoder”). The sign-invariance module operates along the paths and obtains sign-invariant features by applying a single MLP independently to each q-vector implementing a mapping ${\mathbb{R}}^{3}\to {\mathbb{R}}^{128}$ . The corresponding signals bypass the sign-invariance module and are concatenated with the sign-invariant features. The transform modules (Fig. 1b, left) also operate along the paths applying a single module-specific MLP independently to each input. For example, the first transform module implements a mapping ${\mathbb{R}}^{128\;+\;1}\to {\mathbb{R}}^{128}$ which is applied to each of the N sign-invariant features and signals. In contrast, the pooling module (Fig. 1b, middle) operates across the paths, has no learnable weights, and aggregates information via across-the-paths max-pooling, for example, ${\mathbb{R}}^{N\;\times \;128}\to {\mathbb{R}}^{128}$ for the first pooling module. The assembly module (Fig. 1b, right) concatenates along-the-paths features from the transform module with the across-the-paths features of the pooling module independently per path, for example, implementing the mapping ${\mathbb{R}}^{128}\times {\mathbb{R}}^{128}\to {\mathbb{R}}^{256}$ in the first assembly module. Because each module accepts an arbitrary number of inputs and each pooling module’s “global features” are invariant to permutations of the paths, these properties extend to the entire encoder. The encoder has a single output: the latent vector $z\in {\mathbb{R}}^{L}$ representing a summary of the N measurements that served as input.

#### 3.1.2 Decoder

The decoder’s task is to predict signals for the M query vectors given the latent summary z of the encoder while keeping the number M flexible (aim 1b) and to implement aim 3. Like the along-the-paths processing in the encoder, the decoder dynamically allocates one processing path per query vector and independently predicts the signal for that vector (see Fig. 1a, “Decoder”). More specifically, for each query vector and independently along each path, the decoder first obtains sign-invariant features with the sign-invariance module implementing a mapping ${\mathbb{R}}^{3}\to {\mathbb{R}}^{128}$ . Then, the assembly module concatenates the sign-invariant features per path with the latent vector z: ${\mathbb{R}}^{128}\times {\mathbb{R}}^{L}\to {\mathbb{R}}^{128\;+\;L}$ per path. In a final step and independently per path, the decoder’s MLP predicts the signal from the concatenated tensor: ${\mathbb{R}}^{128\;+\;L}\to \mathbb{R}$ .

#### 3.1.3 Sign invariance module (SI)

To implement antipodal symmetry, we introduce a sign-invariance module at the start of both the encoder and decoder for the observation and the query vector, respectively. With SI(q)=F(q)+F(−q) guaranteeing the invariance to the sign of q generally, our sign-invariance module implements a multi-layer perceptron (MLP) for F via $\text{SI(}q\text{)}={\text{MLP}}_{\text{SI}}(q)+{\text{MLP}}_{\text{SI}}(-q)=\text{SI(}-q\text{)}$ as suggested by Lim et al. (2023). In practice, we share the weights of the ${\text{MLP}}_{\text{SI}}$ across all instances in the encoder and decoder.

#### 3.1.4 Details

Each of the MLP layers in the transform modules is composed of a fully connected linear layer, a batch-normalization (Ioffe & Szegedy, 2015), and a hyperbolic tangent activation. The MLP layers in the sign-invariance module and the decoder consist of a fully connected linear layer and a ReLU activation. The last layer of the decoder is only a linear layer. The MLPs in the sign-invariance module contain three hidden and one output layer whereas the remaining MLPs contain two hidden and one output layer. With two exceptions, all layers have a dimension of 128. The size of the output layer of the final transform module in the encoder, that is, the length of the latent vector, is determined via ablation in Section 5. The output layer of the decoder MLP has dimension 1, yielding one scalar signal prediction per query vector.

### 3.2 Augmentations

To achieve invariance with respect to rotations in q-space, we sample random rotations uniformly (Shoemake, 1992) and apply them jointly to observation and query vectors during training. To learn the reconstruction from an arbitrary choice of observation vectors, we input a random choice of at least five observation vectors. To implement this “per-voxel sub-sampling” in fixed-shaped tensors, we input all observation vectors jointly with a binary mask indicating which vectors should be used for reconstruction (see also Appendix Fig. A). This mask is applied within each pooling operation in the encoder. At inference time, we input only the sub-sampled observation set. Both augmentations are applied per training sample providing a diversity of q-space sampling and rotations within each batch.

Since we have normalized the diffusion signal, we can assume that the signal at q≡0 is 1. Therefore, we also query q≡0 during training and obtain a loss for the discrepancy between prediction and target value equal to 1.

### 3.3 Loss function

To guide network training, we use the mean squared error between the signal predictions $\hat{s}$ and their corresponding reference signals s. Additionally, we partition signals depending on whether they are part of the observation set (denoted O) or not (denoted Q\O ) and weigh their relative error contributions with the parameter λ, which we determine in the ablation section.

$$ℒ=(1-\lambda )\;\;\sum_{s\in O}{\left(\hat{s}-s\right)}^{2}+\lambda \;\;\sum_{s\in Q\backslash O}{\left(\hat{s}-s\right)}^{2}$$

## 4 Experiments

This section details the datasets, the training, and the reference methods for the method comparison in the Results section. Finally, we introduce the quality metrics for the diffusion signals and signal-derived measures evaluations.

### 4.1 Datasets

#### 4.1.1 Human Connectome project (HCP)

This dataset features 270 DWIs acquired at diffusion weightings (b-values) of 1,000, 2,000, and 3,000 s/mm^2^ with 90 DWIs per b-value and participant on a modified 3 Tesla Siemens MAGNETOM Skyra scanner (Feinberg et al., 2010; Moeller et al., 2010; Setsompop et al., 2012; Sotiropoulos et al., 2013; van Essen et al., 2012; Xu et al., 2012). Further parameters were: 1.25 mm isotropic spatial resolution, slice multiband factor 3, TR=5,520 ms , TE= 89.5 ​​​ms , Δ=43.1 ​​​ms , and δ=10.6 ​​​ms. We build gender-balanced training-, validation-, and test-sets featuring data from 8, 2, and 20 participants, respectively. From this dataset, we form a multi-shell dataset and a single-shell dataset (featuring signals on the b=1,000 s/mm^2^ shell).

#### 4.1.2 Rhineland Study (RLS)

We utilize data with a grid sampling acquired in the Rhineland Study (Breteler et al., 2014; Stöcker, 2016). This acquisition features DWIs for 112 q-vectors sparsely sampled (Tobisch et al., 2018) from an 11  × 11×11 Cartesian grid in q-space with b-values in the range of 270 to 6,800 s/mm^2^. All images were obtained on a 3 Tesla Siemens MAGNETOM Prisma scanner at an isotropic spatial resolution of 1.5mm , a multiband factor of 3, and with scan parameters TR=5,500ms , TE=105ms , Δ=51.3ms , and δ=20.1ms . We build gender- and age-balanced training-, validation-, and test-sets featuring data from 16, 2, and 20 participants.

#### 4.1.3 High-SNR dataset

For one healthy young male participant and RLS parameters, we acquired DWIs for the RLS q-space sampling and additional low-noise DWIs (20 times repeated measurement of 20 q-vectors, repeated measurements are averaged). Six b-vectors uniformly cover the shell at 1,000 s/mm^2^, while 15 vectors are contained in the Rhineland Study protocol and have been chosen to uniformly cover the b-value range up to 6,800 s/mm^2^. These images were acquired on a 3 Tesla Siemens MAGNETOM Prisma scanner with approval from the ethics committee of the Medical Faculty, University of Bonn, Germany, and with written, informed consent of the participant.

#### 4.1.4 Dataset processing

While the HCP dataset is distributed as preprocessed data, we account for off-resonance fields, eddy currents, and head motion in the Rhineland Study data using *FSL topup* (Andersson et al., 2003; Smith et al., 2004) and *eddy* (Andersson & Sotiropoulos, 2016). Since *eddy* expects the q -vectors to be located on shells, we approximate each q-vector by its nearest neighbor on one of 20 shells as outlined by Tobisch et al. (2018). Furthermore, to address T2-shinethrough, we normalize DWIs by dividing them with the mean q≡0 image of the respective participant. We constrain the scope of training and evaluation to white and grey matter voxels based on tissue segmentations obtained with *FSL FAST* (Smith et al., 2004; Y. Zhang et al., 2001). Cerebrospinal fluid, air, and voxels with a mean q≡0 intensity of zero are excluded.

#### 4.1.5 Observation and query sets for evaluation

For each dataset, we first obtain one set of 30 query vectors and then multiple sets of observation vectors, which do not overlap with the query set. For a meaningful evaluation, we require each observation vector set and the query vector set to cover the respective domain (spheres or 3D space) as uniformly as possible. We ensure the best uniform coverage for the query set by drawing it first from all available vectors. From the vectors not contained in the query set, we draw a nested list of observation sets containing N=10, 20, 30, 50, (80, 160) vectors starting with the largest set. For the shell-based datasets, we obtain these sets via *DMRITool* (Cheng et al., 2018) yielding sets with uniform radial (constrained to shells) and angular coverage (also across shells). Aiming to maintain a consistent angular and radial distribution of vectors for the grid-based dataset, we employ the following approach: First, we group vectors by b-value range (using bins of size 1,000 s/mm^2^). Second, we sort the vectors in each group by choosing a random starting vector and then successively adding the vector with maximum spherical distance to the already chosen set of vectors until no vectors are left. Finally, we draw N samples evenly spaced from the obtained list.

### 4.2 Training implementation

Our method is trained and evaluated on an NVIDIA V100 GPU with 32 GB memory and a batch size in the range between 4,000 and 10,000 depending on the dataset. The learning rate (initially 0.01 ) is reduced (i.e., multiplied by 0.1 ), whenever the loss on the validation set has plateaued for 20 epochs. Early stopping is applied after a minimum of 40 epochs when the validation error has not decreased for 40 successive epochs. As described in Section 3.2, we sample independent observation sets by random undersampling during training. The upper ceiling of the observation vectors is given by the measurements available in each dataset, that is, 270 for the HCP multi-shell dataset, 90 for the HCP single-shell dataset, and 112 for the grid-based dataset (see Section 4.1).

### 4.3 Reference methods

Inspired by reconstruction challenges (Ning et al., 2015, 2019), we compare our method against the following state-of-the-art methods: For the reconstruction of signals on a spherical domain, that is, for single-shell acquisitions, we choose the regularized least squares fit of spherical harmonics functions (Descoteaux et al., 2007) using the *Dipy* implementation (Garyfallidis et al., 2014) with an order of 8 and refer to this as *SH*. For reconstruction of signals on a continuous 3D space, that is, for multi-shell or grid-based acquisition, we use the *Dipy* implementations of *SHORE* (Özarslan et al., 2009) and *MAP-MRI* (Özarslan et al., 2013) (radial order 4 consistent with the setting in Ning et al. (2015)). We also compare with a compressed sensing approach (Paquette et al., 2015; Tobisch et al., 2018, 2019) (*CS*) on the grid-based dataset. Here, the optimization problem is solved via the iterative shrinkage and thresholding algorithm (Gong et al., 2013). On the validation set, we determine the optimal values for the regularization parameter $\lambda_{\text{CS}}$ via grid-search (between 1.0 and $10^{-9}$ ): $\lambda_{\text{CS}}=5\cdot 10^{-6},5\cdot 10^{-6},$ $1\cdot 10^{-6},1\cdot 10^{-6},5\cdot 10^{-7}$ for 10, 20, 30, 50, and 80 observation vectors, respectively.

In addition, we compare with discrete learning-based methods (see Section 2) for all domains. All discrete learning-based methods are based on MLPs predicting some signal-derived metric. For a signal reconstruction comparison, we adapt the network by Golkov et al. (2016) adjusting the output layer of the MLP to predict all signals available at training time. To ensure a fair comparison, we improve and ablate the best-performing MLP architecture under the condition of a similar number of parameters as *DISCUS* (≈170k parameters). The ablation, for example, establishes the optimal allocation of parameters to network depth (number of layers) and breadth (units per layer). We chose the best-performing approach on the validation set—an MLP having one hidden layer with 566 units—followed by a ReLU activation for this reference method and refer to it as *MLP*. We trained one instance per observation set for each dataset (a total of 15 networks) using stochastic gradient descent with a momentum of 0.9. The learning rate scheduling and early stopping are applied analogously to our method.

We also compare with a learning-based approach that learns SH-coefficients providing similar query flexibility for single-shell sampling as *DISCUS* (*MLP-SH*). This method predicts SH-coefficients from a signal vector. Based on work by Ye (2017), observations, and our ablation, we choose an end-to-end model driven by a signal reconstruction loss instead of a coefficient reconstruction loss. This end-to-end training also minimizes the previously mentioned information-loss limitation. More specifically, we reconstruct signals based on SH-coefficients during training and compute the $\ell_{2}$-loss between predicted and reference signal vectors. The network is the same as the aforementioned *MLP* but has a fixed number of 45 output neurons representing the SH-coefficients with order 8 and an initial learning rate of 0.01. As the number of input neurons is fixed and tied to the observation set, separate instances, that is, one per observation set, are trained analogously to the *MLP* method.

### 4.4 Objects of analysis and evaluation metrics

We compare different methods with respect to the quality of their respective signal reconstructions as well as the outputs of some typical analyses of these signals. As each voxel is an independent sample, we compare distributions of voxel-wise error metrics across the voxels of the test participants. These distributions are represented by boxplots, where the box is bounded by the 25th and 75th percentile; the marker inside the box denotes the median; the whisker’s extension from the box denotes minimum (maximum) of 1.5 times the interquartile range (height of the box) and the maximum (minimum) error. For signal predictions ${\hat{s}}_{ij}$ and reference signals $s_{ij}$ at voxel i and for q-vector j=1,...,J , we quantify the reconstruction error via

$$Error_{\text{signal}}({\hat{s}}_{i},s_{i})=\frac{1}{J}\sum_{j=1}^{J}\frac{{\left({\hat{s}}_{ij}-s_{ij}\right)}^{2}}{s_{ij}^{2}}\text{.}$$

We have evaluated our method on various error metrics (squared, normalized squared, absolute error) and different ways to aggregate errors per imaging voxel, that is, along diffusion signals per voxel (mean, median, percentiles), but both the ranking and the overall statistics are consistent across different error metrics.

Furthermore, we compare reconstruction methods on downstream analyses of the signal by reconstructing DWIs from the observation sets and fitting *Diffusion Tensor Imaging* (DTI) (Basser et al., 1994; Westin et al., 1997), *Diffusion Kurtosis Imaging* (DKI) (Jensen et al., 2005), *Neurite Orientation Dispersion and Density Imaging* (NODDI) (H. Zhang et al., 2012), and *Mean Apparent Propagator MRI* (MAP-MRI) (Özarslan et al., 2013) models. As an additional baseline, we also compare to the model-fits on the observation sets, that is, without intermediate reconstruction of withheld q-space signals.

We fit the DTI model with *FSL dtifit* (Smith et al., 2004), the DKI and MAP-MRI models with *Dipy* (Garyfallidis et al., 2014), and the NODDI model with the *Microstructure Diffusion Toolbox* (Harms & Roebroeck, 2018; Harms et al., 2017). We quantify the error between predicted metrics ${\hat{m}}_{i}$ with metrics obtained from model-fits on the full reference data $m_{i}$ at voxel i via

$$Error_{\text{metric}}({\hat{m}}_{i},m_{i})=\frac{{\left({\hat{m}}_{i}-m_{i}\right)}^{2}}{m_{i}^{2}}\text{.}$$

For the tensor model, we obtain separate reference data per observation set, that is, 10, 20, 30, etc. by fitting the tensor model only to those measurements not contained in the observation set, that is, the remaining 80, 70, 60, etc. to make input observation and output tensor fit independent and to avoid rewarding overfits to noise in the observation set.

Finally, we compare angular errors of peaks of fiber orientation distribution functions (fODFs) obtained via constrained spherical deconvolution on the single-shell data of the HCP dataset with the *MRtrix* package (Tournier et al., 2019). More specifically, we obtain response functions with *dwi2response* (Tournier et al., 2013), ODFs with *dwi2fod* (Tournier et al., 2007), and extract three peaks of the ODFs via *sh2peaks* (Jeurissen et al., 2013). We obtain masks for three characteristic fiber configurations: single fiber, crossings of two and three fibers (see Appendix Section A.3 for details) and report the distribution of average angular errors over voxels for each configuration and different observation set sizes.

## 5 Ablation

To explore the impact of specific method components on performance on the data of the validation participants, we run these ablations by varying a single method component of interest and keeping everything else fixed. While we tested various components of our method, including different encoder and decoder designs, loss functions, and hyper-parameters, we present only five ablations in this section: latent space dimension and number of training participants on the HCP multi-shell dataset, augmentation schemes on the HCP single-shell dataset, and the ablation of loss function components on both datasets.

### 5.1 Latent space

**Fig. 2.**
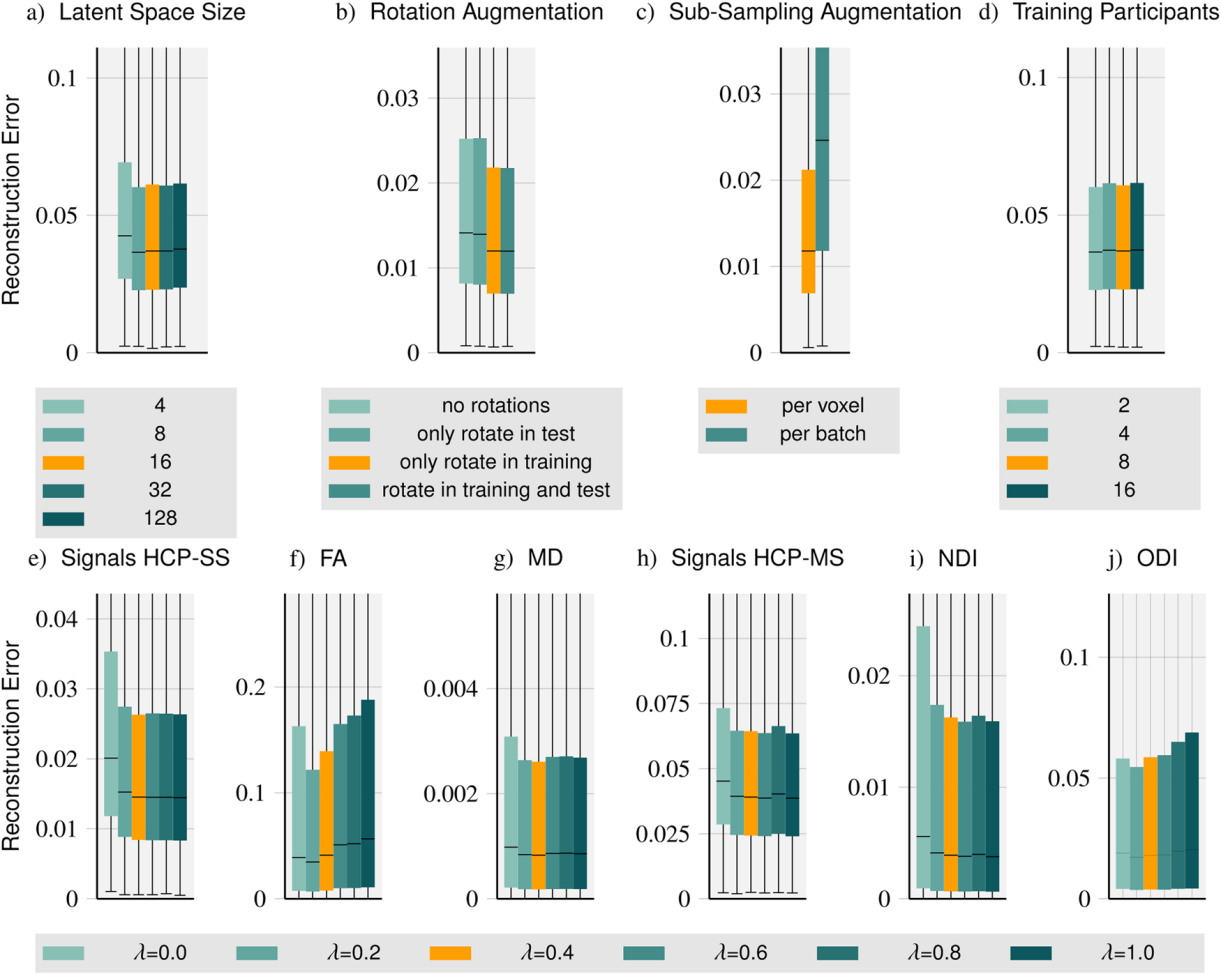
Method ablations: We compare the impact of the latent space dimension (a), rotation augmentation (b), sub-sampling augmentation (c), and the number of training participants (d) on method performance. Furthermore, we vary the loss function parameter λ and evaluate signal reconstructions for single- and multi-shell (HCP-SS (e) and HCP-MS (h), respectively) as well as tensor-derived FA (f) and MD (g) based on HCP-SS data and NODDI-derived NDI (i) and ODI (j) based on HCP-MS data.

The latent space is the interface between the encoder and the decoder. Its dimension determines the amount of information that can be passed between the two components. To compare the role of the latent space dimension on performance, we train separate instances of our method with latent space dimensions of 4, 8, 16, 32, and 128 illustrated in Figure 2a. We find that the performance is best for a latent space of dimension 16 which is close to the optimal value for the compression of the diffusion signal with autoencoders determined by Zucchelli et al. (2021). In contrast, for small latent spaces, performance is substantially worse, suggesting that a dimension of 16 is large enough for sufficient information to be passed between the encoder and the decoder. Unsurprisingly, the full, rich information in dMRI cannot be compressed into a very small latent space. The performance decline for larger latent spaces is typical for autoencoder designs as compression is often a critical feature to regularize the reconstruction.

### 5.2 Rotation augmentation

As outlined in Section 3.2, we apply random rotations to the diffusion-encoding vectors during training. To show the effect of this augmentation type, we train two instances with and without rotation augmentation and evaluate each instance with and without a random rotation at test time. In Figure 2b, we compare the four instances. Comparing the model instances with rotation augmentation during training, we see that the rotation at test time does not impact performance at all, as expected. Performance improves a little when a model trained without rotations is tested on rotated data. Omitting the rotation augmentation during training worsens performance by about 17% while a bigger performance loss would be expected. This is likely because diffusion-encoding vectors of the same acquisition vary very little between participants and the coverage of q-vectors on the sphere is dense (90 vectors and mirrored versions, with inter-participant variance).

### 5.3 Sub-sampling augmentation

In addition to diverse rotations, we sub-sample and dynamically mask the observation set to establish diverse observation sets within each batch (“per voxel”, see also Section 3.2). As an alternative approach, we compare to a single random sub-sampling “per batch”. Figure 2c shows dynamic, diverse, “per voxel” sub-sampling significantly outperforming “per batch” sub-sampling underlining the impact of signal sampling diversity even within a batch.

### 5.4 Training participants

We compare performance when using data from different numbers of training participants. We evaluate separate method instances trained with data from 2, 4, 8, and 16 participants, respectively. As shown in Figure 2d, the number of training participants surprisingly has no substantial impact on method performance. Since the reconstruction in each voxel is considered an independent reconstruction task, we attribute the performance to the number of training samples per participant (approximately 400,000 voxels are contained in the white and grey matter mask per HCP participant).

### 5.5 Loss weighing λ

In a final ablation, we explore the impact of the parameter λ weighing the contributions of observation and query components in the loss function on performance. In contrast to previous results, here, we analyze the errors for signal prediction and signal-derived microstructure models (DTI, NODDI). For signal prediction in single- and multi-shell settings, larger values of λ, that is, higher emphasis on the prediction of (unseen) signals (not contained in the observation set) improve performance. We observe similar behavior for *Mean Diffusivity* (MD) and the *Neurite Density Index* (NDI). In contrast, for *Fractional Anisotropy* (FA) and the *Orientation Dispersion Index* (ODI), we observe an almost opposite characteristic showing a performance decrease for higher values of λ. Small values of λ incentivize the reconstruction of (noisy) inputs only which are possibly just passed through the network while the training of signal reconstruction for unseen query vectors is very limited. For larger values of λ, the incentive is shifted towards the signal prediction on uniform samples of q-space where extreme values essential for accurate FA estimation are rarely attained. Taking the two patterns into account, we choose λ=0.4 yielding a mixture of observation and query components in our method’s loss function.

## 6 Results

This section contains two parts, focusing on different aspects of reconstruction methods. First, we compare our *DISCUS* method against established state-of-the-art methods and compare the signal reconstruction quality on HCP and RLS datasets with different q-space samplings. Then, we compare reconstruction methods on signal-derived microstructure models frequently used for the analysis of dMRI signals.

### 6.1 Signal reconstruction

**Fig. 3.**
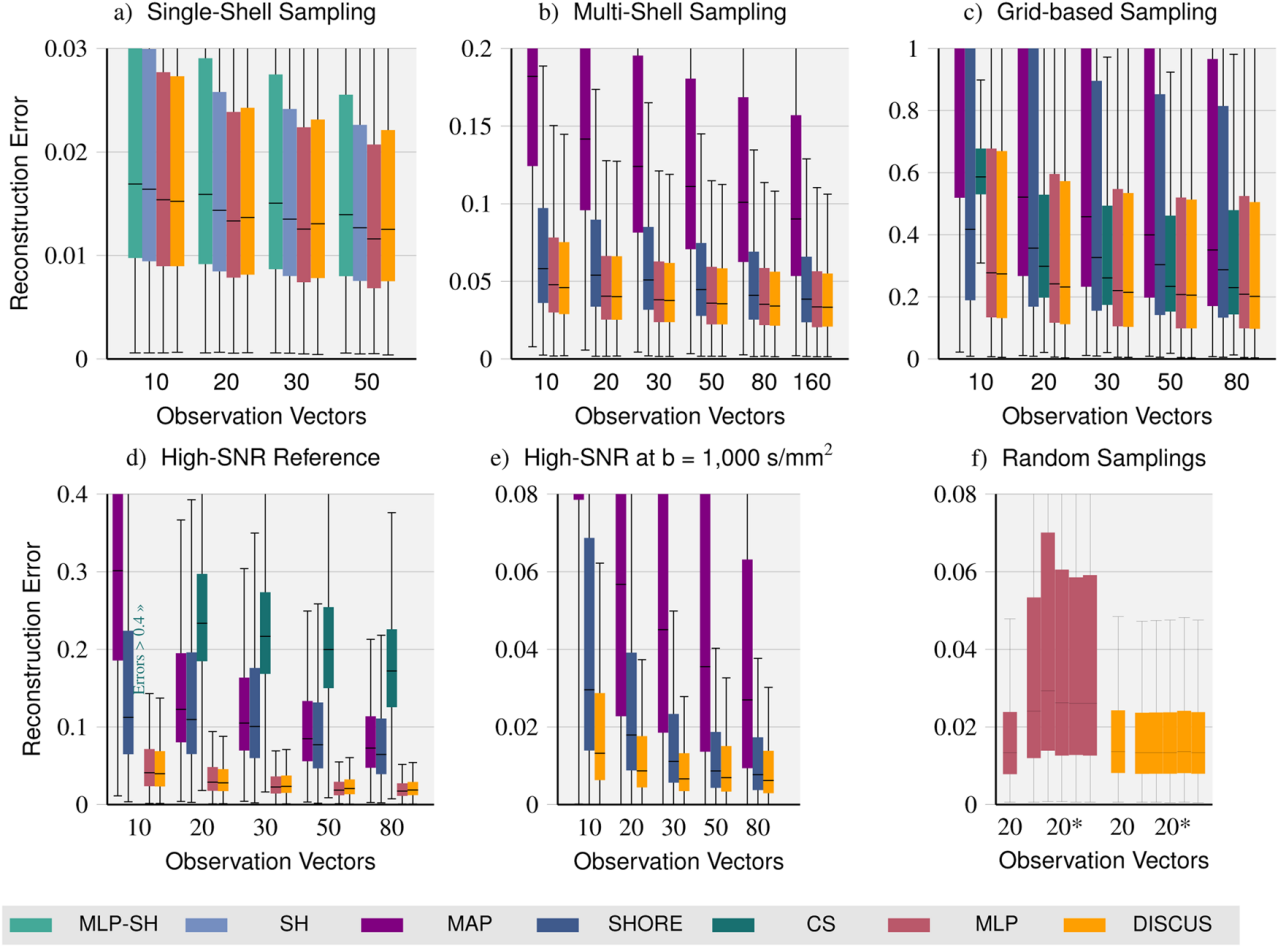
Quantitative signal reconstruction comparison: On datasets featuring a single-shell (a), a multi-shell (b), and a grid sampling of q-space (c), we compare our *DISCUS* method (yellow) with state-of-the-art reconstruction methods: an MLP learning spherical harmonics coefficients (*MLP-SH*), spherical harmonics (*SH*) (on single-shell data), MAP-MRI (*MAP*), *SHORE* on multi-shell and grid-based data, and compressed sensing DSI (*CS*) on grid-based data as well as the *MLP*, which is separately trained per dataset and sub-sampling. For all methods, we obtain decreasing reconstruction errors for larger observation sets as more information for reconstruction is available. The learning-based methods *MLP* and *DISCUS* seem to outperform *SH*, *MLP-SH*, *CS*, and *SHORE* slightly and considerably so for *MAP*. Both the *MLP* and *DISCUS* yield almost equal error distributions. Other aspects of the signal reconstruction method comparison: We compare the reconstruction quality on two high-SNR reference datasets: The first set (d) contains high SNR signals for q-vectors contained in the sampling scheme of the Rhineland Study. The second set (e) contains six high-SNR signals for q-vectors at a b-value of 1,000 s/mm^2^ which are not contained in the Rhineland Study sampling (training data). Unlike the *MLP* and *CS*, *SHORE*, *MAP*, and our *DISCUS* method support the prediction of signals for q-vectors not contained in the training data. Subfigure (f) compares the *MLP* and *DISCUS* networks trained on the single-shell dataset on different subsets of 20 observation vectors: First, on the predefined set of Subfigure (a), and then additionally on five unseen, randomly sampled observation sets (20*). None of the observation vectors are contained in the query set. As the random observation sets do not match the training data, performance drops for the *MLP* method whereas our *DISCUS* method maintains its performance. Parts (e) and (f) emphasize the capabilities *DISCUS* has over the *MLP* method.

Here, we compare *DISCUS* with state-of-the-art reference methods (see Section 4.3) on datasets with single-shell, multi-shell, and grid-sampling of q-space (see Section 4.1). On the single-shell dataset (see Fig. 3a), we compare our method with the *MLP-SH*, *SH*, and *MLP* methods. The *SH* method is fit independently to each set of observation vectors to provide signal predictions for the corresponding query vectors. The *MLP* requires separate training and a dedicated model per set of observation vectors, that is, 4, 6, and 5 models for single-shell, multi-shell, and grid, respectively. Analogously, four separate *MLP-SH* instances are trained for the single-shell dataset. In contrast, *DISCUS* only uses one model per sampling paradigm.

For all methods, we observe decreasing errors with larger observation sets indicating the increasing benefit methods can gain from the increased, available information for reconstruction. For larger observation sets, the reconstruction quality of *SH* is equivalent to our *DISCUS* method. For smaller observation sets, we note a gap where our method outperforms *SH*. The extremely similar error distributions observed for *MLP* and *DISCUS* across observation sets illustrate that the error is increasingly dominated by the acquisition noise. For larger observation sets, we note a gap where the *MLP* has slightly lower errors compared to *DISCUS*. All methods outperform *MLP-SH* on all observation sets.

On the multi-shell dataset (see Fig. 3b), we compare *DISCUS* with the *MLP*, *MAP*, and *SHORE* methods. Analogously to *SH*, *MAP* and *SHORE* are fit independently to each observation set, voxel, and participant. Here, we also observe decreasing errors for larger observation sets for all methods. Both *MAP* and *SHORE* are outperformed by the *MLP* and *DISCUS* across observation sets. The error distributions of *MLP* and *DISCUS* look very similar with slightly lower errors for *DISCUS*.

Finally, for the grid-based sampling, we compare our approach with *MAP*, *SHORE*, *CS*, and the *MLP* (see Fig. 3c) finding decreasing errors for all methods for larger observation sets. Similar to the multi-shell dataset, the learning-based methods *MLP* and *DISCUS* outperform both *MAP* and *SHORE* substantially. For the *CS* method, we note narrower error distributions. However, while its performance is very good for large observation sets, the performance declines slightly for smaller observation sets and drops substantially when only 10 observation vectors are available. As on the multi-shell dataset, *MLP* and *DISCUS* have very similar error distributions with a slight advantage for *DISCUS*.

As the acquisition noise is a substantial part of the errors, reconstruction methods are evaluated and compared on the high-SNR evaluation dataset (see Fig. 3d and e). Here, *MAP*, *SHORE*, *CS*, *MLP*, and *DISCUS* methods are fit to the same observation sets as in the Rhineland Study evaluation above. However, reconstruction quality is determined on the high-SNR signals based on 20 acquisitions.

In the first part of this evaluation, these high-SNR signals were obtained for q-vectors also contained in the Rhineland Study, that is, the *CS* and pre-trained *MLP* methods are applicable. We observe low performance of *CS*, performance in the middle range of *MAP* and *SHORE*, and high performance of the pre-trained learning methods *MLP* and *DISCUS* which have very similar error distributions again.

In the second part, we evaluate signals for q-vectors not contained in the sampling of the RLS, that is, unknown for the training dataset where the *CS* and *MLP* methods cannot be applied. Figure 3e shows that *DISCUS* substantially outperforms *SHORE* and *MAP*.

Finally, we test the effect of changes to the observation sets at test time and compare the resulting performance on the single-shell dataset in Figure 3f. As expected, the performance of the *MLP* suffers a lot as the choice and order of observations differ from the fixed pattern used for training. In contrast, our *DISCUS* method maintains its performance as it is permutation-invariant by design and has been trained to reconstruct based on arbitrary observation sets. Here, the remaining variability in performance between observation sets stems from the fact that different observation sets contain different acquisition noise and information for the reconstruction.

### 6.2 Downstream analysis models

**Fig. 4.**
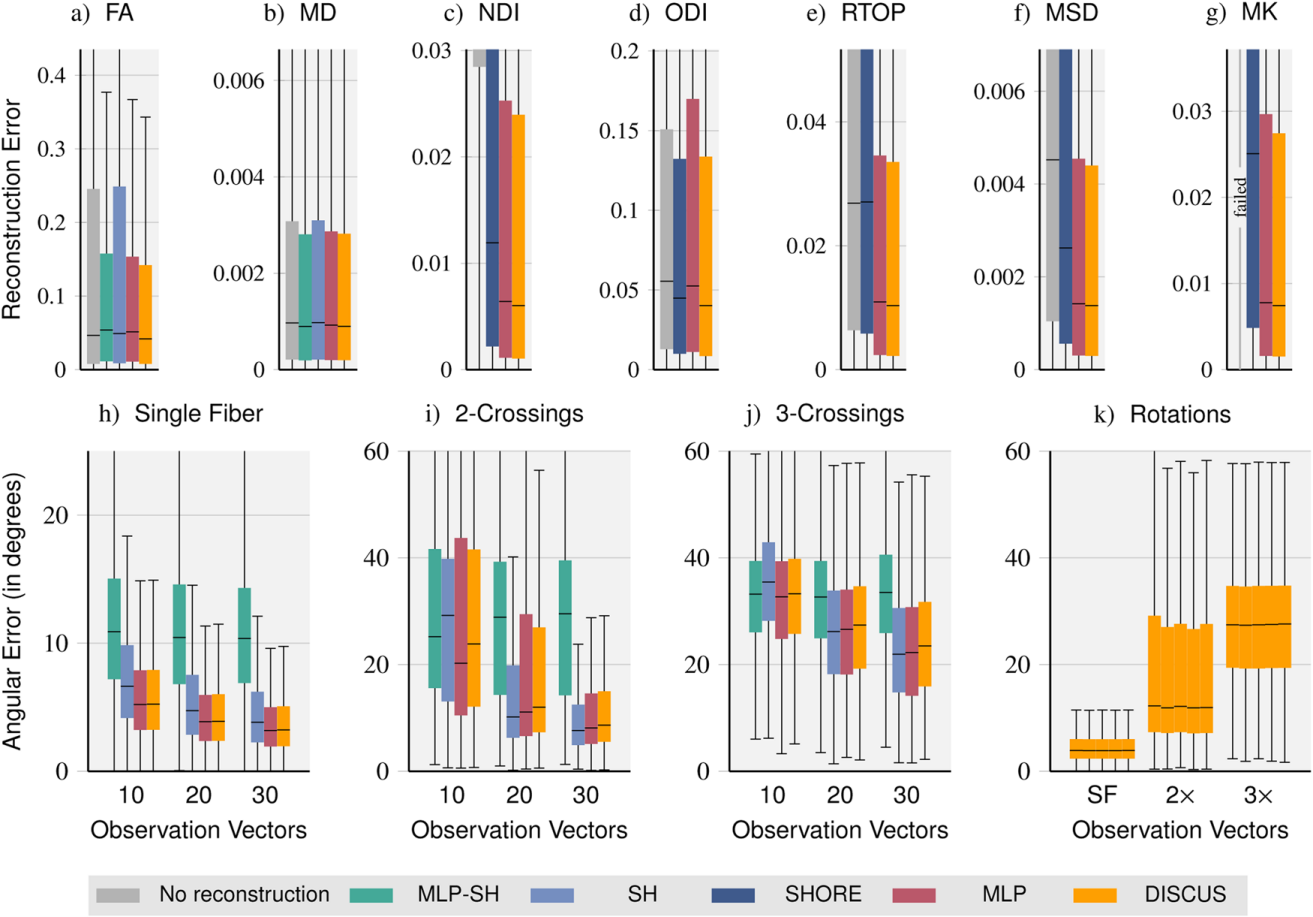
Method comparison on signal-derived metrics: We compare the quality of DTI-derived *Fractional Anisotropy* (FA), *Mean Diffusivity* (MD), NODDI-derived *Neurite Density Index* (NDI) and *Orientation Dispersion Index* (ODI), MAP-MRI-derived *Return To Origin Probability* (RTOP) and *Mean Squared Displacement* (MSD), and DKI-derived *Mean Kurtosis* (MK) metrics obtained with sub-sampled data (no reconstruction) and with data reconstructed with the *MLP-SH, SH, SHORE, MLP,* and *DISCUS* methods, respectively (a-g). In addition, we compare the angular errors of fiber orientation distribution function (fODF) peaks for different fiber configurations: single fiber (h), and crossings of two (i) and three fibers (j), respectively. Finally, we let *DISCUS* reconstruct signals for five random rotations in q-space, obtain fODF peaks, and report angular errors for single fiber (SF), two- and three-crossings (2× and 3× ) illustrating the rotation invariance of *DISCUS* (k).

Beyond signal reconstruction, we evaluate how signal predictions impact measures derived from the DWIs. In Figure 4, we compare the reconstruction methods on DTI-derived metrics for observation sets with 10 observation vectors of the single-shell data and DKI-, NODDI-, and MAP-MRI-derived metrics based on 10 observation vectors of the multi-shell data. Target reference values are computed from all DWIs not contained in the observation set, that is, 80 DWIs for the single-shell, but all 270 DWIs for the multi-shell data.

For the DTI-derived metrics *Fractional Anisotropy* (FA) and *Mean Diffusivity* (MD), we compare fits directly on the sub-sampled data (no reconstruction of missing signals) and 90 signals reconstructed with *MLP-SH*, *SH*, *MLP*, and *DISCUS*, respectively. For FA, the learning-based methods *MLP-SH*, *MLP* and *DISCUS* outperform *no reconstruction* and *SH*. *DISCUS* performs best. For MD, we obtain very similar error distributions overall and particularly for *MLP-SH*, *MLP*, and *DISCUS*.

On the multi-shell dataset, we compare methods on DKI, NODDI, and MAP-MRI models. Due to the bad signal reconstruction performance of *MAP* on the multi-shell dataset, we exclude *MAP* in this comparison. For the *Neurite Density Index* (NDI), *no reconstruction* performs substantially worse indicating that the reconstruction of additional signals is very beneficial for this metric. Again, the learning-based methods perform best. For the *Orientation Dispersion Index* (ODI), *SHORE* outperforms *no reconstruction* and *MLP*, while *DISCUS* outperforms *SHORE* slightly. Similarly, for the *MAP-MRI*-derived metrics *Return To Origin Probability* (RTOP) and *Mean Squared Displacement* (MSD) and DKI-derived *Mean Kurtosis* (MK), the learning-based methods outperform *SHORE* and *no reconstruction*. Without reconstruction, the diffusion kurtosis model failed, that is, returned only zeros when only 10 observation vectors were available. On closer inspection, we saw a sharp performance decline, that is, more and more zeros were returned as we incrementally lowered the number of observation vectors. For most metrics, the results suggest that *DISCUS* performs best albeit just slightly better than the *MLP*.

Another important application of dMRI is the computation of *Fiber Orientation Distribution Functions* (fODFs), for example, to inform tractography. In Figure 4h-j, we evaluate the impact of signal reconstruction with different methods on the estimation of fODF peaks and report error distributions for each method, different degrees of sparsity (10, 20, and 30 observation vectors), as well as different fiber configurations, that is, voxels containing a single fiber and two- and three-crossings. *MLP-SH* is outperformed with very few exceptions. On single-fiber voxels, *MLP* and *DISCUS* perform equally well outperforming *SH*. On voxels with two- and three-crossings, *MLP* and *DISCUS* outperform *SH* for sparse observations (10 q-vectors) and *SH* slightly outperforms the two on larger observation sets (20 and 30 vectors). *MLP* outperforms *DISCUS* on the voxels featuring fiber crossings albeit just slightly in most settings. The magnitude of angular error suggests that fiber directions can be reliably inferred in single-fiber voxels even from sparse acquisitions. In contrast, as the fiber configurations get more complex, more measurements seem necessary to accurately infer the peaks. For two-crossings, we observe lower errors starting at 30 observation vectors. For three-crossings, all methods yield errors above 20 degrees (median value), suggesting that more than 30 vectors are required to resolve three-crossings well. In Figure 4k, we obtain five random rotations of q-space, reconstruct signals with *DISCUS* for the rotated observation and query vectors, and determine fODF peaks. The very similar performance across rotations illustrates the rotation invariance of the *DISCUS* method.

## 7 Conclusion

In this paper, we have presented the first entirely continuous learning-based method for the reconstruction of dMRI signals. This method is input-continuous—it can be applied to any (sufficiently dense) acquisition scheme—and output-continuous—it can infer signals for arbitrary q-vectors. *DISCUS* offers a methodological framework to address two challenges in dMRI: the sparsity of q-space sampling to keep scan times short and the diversity of sampling schemes between studies. Our approach permits the reliable inference of additional signals, especially from short acquisitions. Specifically, we have shown that *DISCUS* outperforms both continuous (*SHORE* and *MAP-MRI*) and discrete model-based methods (*CS*) as well as learning-based semi-continuous methods (*MLP-SH*). Compared to the strongest competitor, an optimized *MLP*, *DISCUS*’s and the *MLP’s* error distributions are very similar, but with the difference that *DISCUS* is entirely continuous offering support for arbitrary samplings—unlike the discrete *MLP*. Additionally, we have demonstrated that *DISCUS*- and *MLP*-derived reconstructions yield very similar errors in downstream evaluations consistently outperforming other reference methods. While technically within error margins, the *MLP* is slightly better in fODF-based angular errors and *DISCUS* is slightly better in estimates of microstructure models (DTI, DKI, NODDI, MAP-MRI). Our proposed *DISCUS* method may be used to accelerate acquisitions, to derive higher-quality signal-derived metrics retrospectively from existing acquisitions, or for dMRI data harmonization by resampling datasets with different q-space acquisition schemes onto a common scheme.

## Figures and Tables

**Appendix Fig. A.**
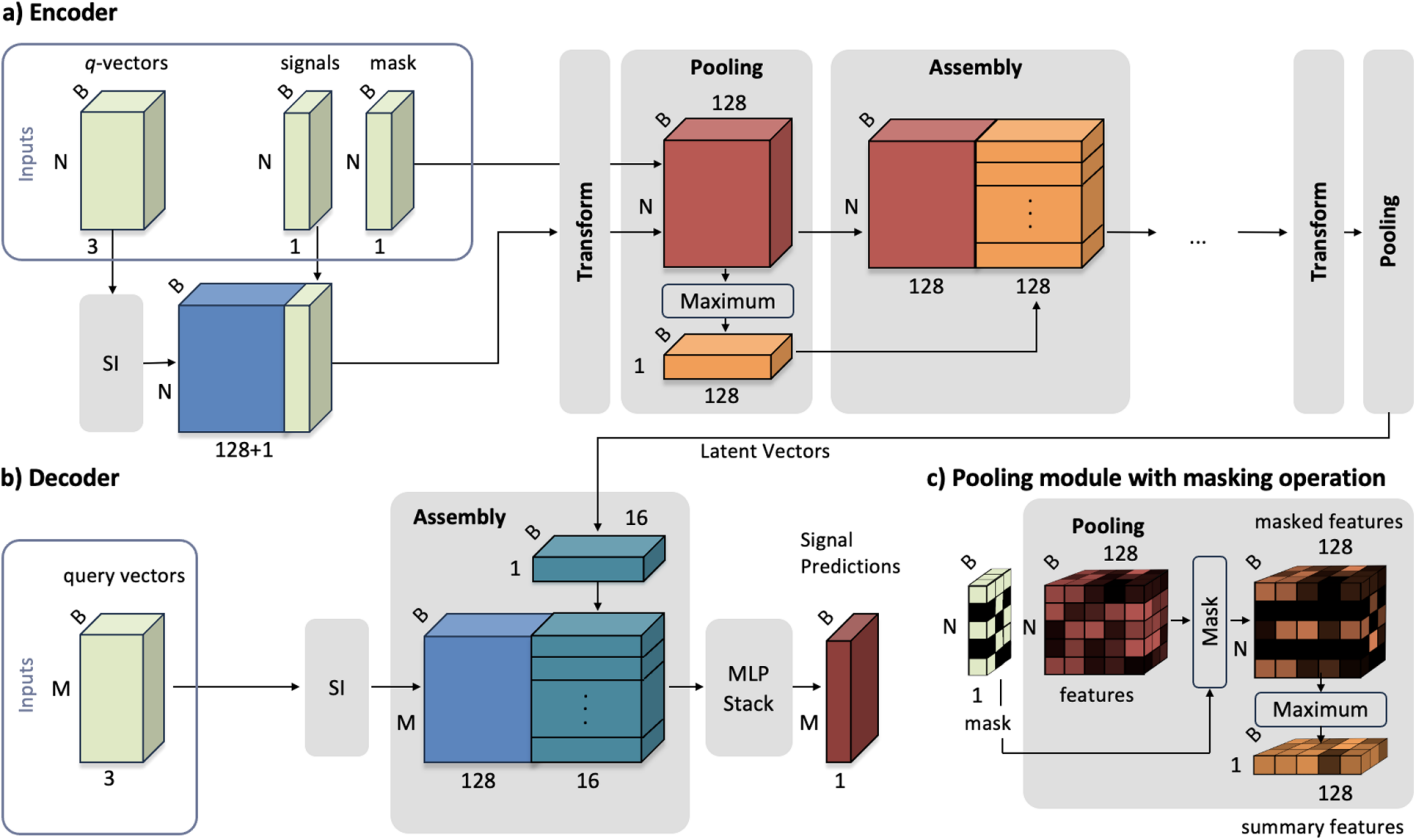
Details of the *DISCUS* architecture: (a) the encoder, (b) the decoder, and (c) the pooling module with masking operation during training. (a) The encoder receives q-vectors (B×N×3 with batch size B and number of observations N), corresponding observed signals (B×N×1 ) and a mask indicating a random subsampling (B×N×1 ). At inference time, the mask may be omitted to deactivate the masking. After sign-invariant features (blue) are obtained from the q-vectors by the *SI* module, the signals are concatenated, and each feature vector of length 128+1 is processed independently by the MLP in the first transform module, resulting in the transform module output (red). Before pooling, the mask is applied to make sure only features for the desired subset of paths are summarized in the pooling operation (see c). Relevant features are summarized (orange), and per-path features (red) are concatenated with summary features (orange) by the assembly module. These are the inputs to the next transform module. The summary features of the final pooling module are the latent vectors (turquoise). (b) The decoder receives M query vectors (B×M×3 ) as input and passes them through the *SI* module obtaining sign-invariant features. An assembly module concatenates the sign-invariant features with the latent vectors to be processed by the MLP into signal predictions (dark red). (c) The masking operation reduces feature values, so masked values are always the smallest in the pooling direction of the proceeding maximum operation. They can thus never contribute to the summarized features.

**Appendix Fig. B.**
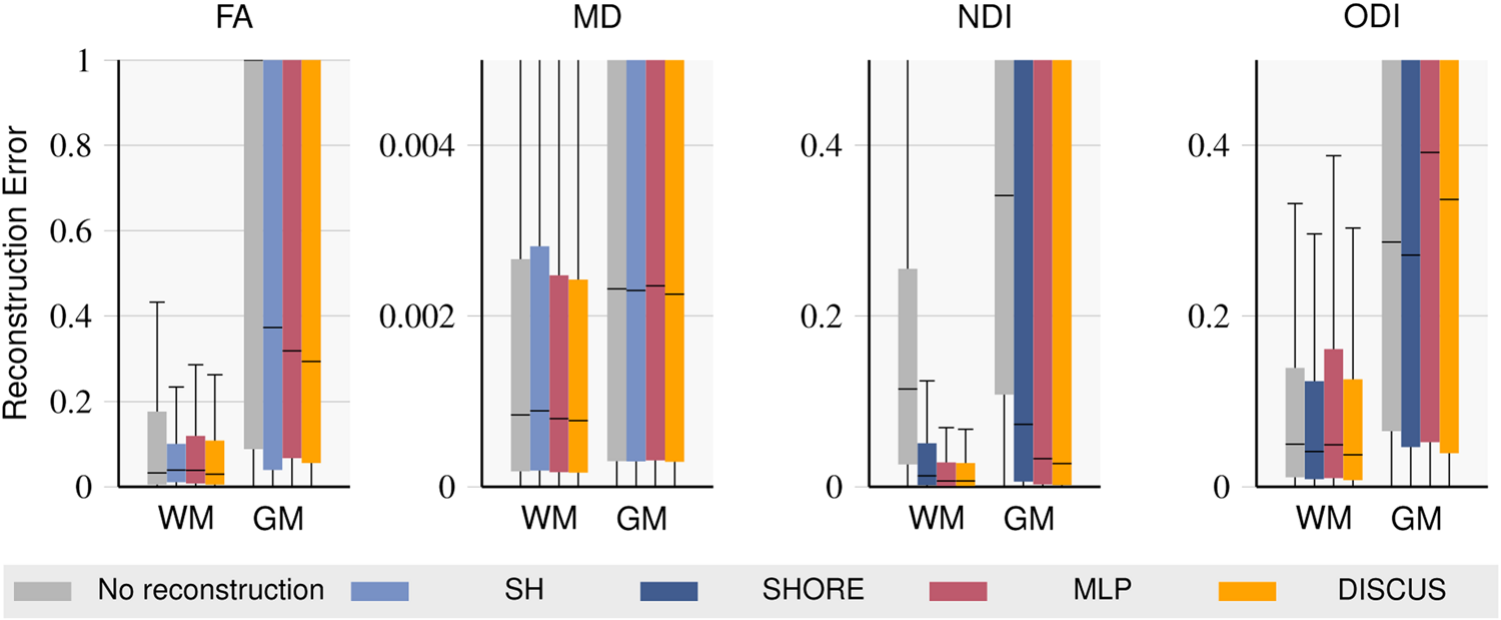
White and grey matter normalized squared errors of the microstructure models derived from reconstructed or sub-sampled data, respectively. While we aggregate errors across grey (GM) and white matter (WM) in our main evaluations, this figure shows the errors per tissue for diffusion tensor-derived Fractional Anisotropy (FA) and Mean Diffusivity (MD) as well as NODDI-derived Neurite Density Index (NDI) and Orientation Dispersion Index (ODI).

**Appendix Fig. C.**
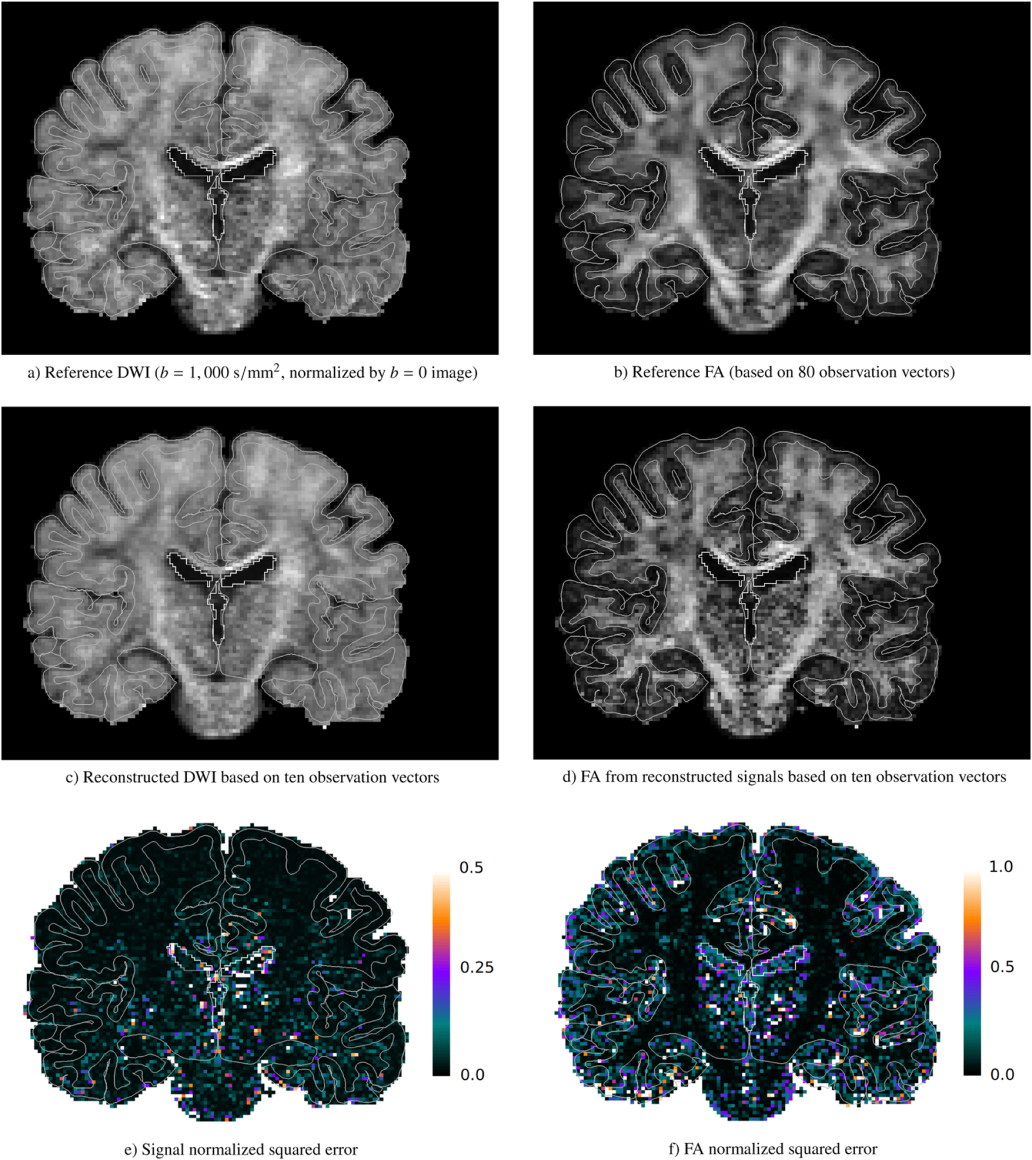
The reference signal and FA maps are shown in (a) and (b). Signals reconstructed with *DISCUS* from only 10 observation vectors and Fractional Anisotropy (FA) based on the reconstructed signals are displayed in (c) and (d). Finally, (e) and (f) illustrate the error maps between reference and predicted maps. Overlaid are the white matter and pial surfaces (gray) as well as the outline of a merged CSF/ventricles region (white). All slices are from or based on the data of the Human Connectome Project (HCP).

## Data Availability

The MRI data of the Human Connectome Project (HCP) are publicly available on the website https://www.humanconnectome.org. The MRI data of the Rhineland Study are not publicly available due to data protection regulations. Access can be provided to scientists in accordance with the Rhineland Study’s Data Use and Access Policy. Requests to access the data should be directed to Dr. Monique Breteler at RS-DUAC@dzne.de. The source code for the *DISCUS* method can be found on the website https://github.com/Deep-MI/DISCUS.

## Appendix

### A.1 Difference maps

In Appendix Figure C, we show an example diffusion-weighted image reconstructed with *DISCUS* from **only 10** observation vectors (for a b-vector that was not part of the observation set). Additionally, we show an FA map computed from 90 predicted DWIs (also predicted based on 10 observations). Along with these images, we plot the reference images and the normalized squared error maps with respect to reference signals and FA maps, respectively. The signal error map shows low errors along major white matter tracts and most of the grey matter. Errors are observed over CSF regions such as the ventricles and are noticeable in the subcortical grey matter. The error in FA is also low along major white matter tracts, pronounced in CSF regions, and substantial in subcortical grey matter, for example, thalamus. Because of the limited relevance of the CSF in dMRI, CSF voxels are not part of the training set and are ignored in the evaluation. Differences in magnitude between white and grey matter and CSF may help explain these error maps (as the error is normalized).

### A.2 Errors in grey and white matter

All reconstruction methods feature larger (normalized) errors in the grey matter compared to the white matter for microstructure model-derived evaluations, such as the diffusion tensor and NODDI fit (see Appendix Fig. B). However, *DISCUS* often outperforms the other reconstruction methods and the approach without reconstruction, illustrating that the difference between GM and WM errors is likely linked to a more complex microstructure in the GM, which requires more DWIs for accurate reconstruction.

### A.3 fODF Evaluation

We evaluate angular errors between peaks of fiber orientation distribution functions (fODF) per fiber configuration, that is, in voxels containing a single fiber, two fibers crossing, and three fibers crossing, respectively. We determine these voxels on the reference data having obtained three (normalized) peaks $p_{1},p_{2},p_{3}$ sorted by their corresponding decreasing magnitudes $m_{1},m_{2},m_{3}$ with angular distances $∡(p_{1},p_{2}),∡(p_{1},p_{3}),∡(p_{2},p_{3})$ . We determine single fiber voxels as having a large peak ($m_{1}>0.5$ ) and two small peaks ($m_{2}<0.2\cdot m_{1}$). Voxels with two-crossings are determined as those with two large peaks ($m_{1}>0.3$ , $m_{2}>0.3$ ) and a small third peak ($m_{3}<0.1\cdot m_{2}$) in addition to a large angular distance between the two large peaks ($∡\left(p_{1},p_{2}\right)>60^{\circ }$). Finally, we determine three-crossings as three similar peak magnitudes ($m_{3}>0.7\cdot m_{1}$, $m_{3}>0.225$ ) with pronounced angular distances between each pair of fibers ($∡\left(p_{1},p_{2}\right)>60^{\circ }$, $∡\left(p_{1},p_{3}\right)>60^{\circ }$, and $∡\left(p_{2},p_{3}\right)>60^{\circ })$ . We filter these sets of voxels and use only connected components of at least 20 voxels for the single fiber voxels and 10 voxels for two- and three-crossings, respectively.
